# A systematic review of empirical bioethics methodologies

**DOI:** 10.1186/s12910-015-0010-3

**Published:** 2015-03-07

**Authors:** Rachel Davies, Jonathan Ives, Michael Dunn

**Affiliations:** C/O Medicine, Ethics, Society and History, School of Health and Population Sciences, The University of Birmingham, 90 Vincent Drive, Edgbaston, Birmingham, B15 2TT UK; Medicine, Ethics, Society and History, School of Health and Population Sciences, The University of Birmingham, 90 Vincent Drive, Edgbaston, Birmingham, B15 2TT UK; The Ethox Centre, Nuffield Department of Population Health, University of Oxford, Rosemary Rue Building, Old Road Campus, Headington, Oxford, OX3 7LF UK

**Keywords:** Empirical bioethics, Method, Methodology, Integrated ethics, Coherence, Consensus, Epistemology

## Abstract

**Background:**

Despite the increased prevalence of bioethics research that seeks to use empirical data to answer normative research questions, there is no consensus as to what an appropriate methodology for this would be. This review aims to search the literature, present and critically discuss published Empirical Bioethics methodologies.

**Methods:**

MedLine, Web of Science and Google Scholar were searched between 15/02/12 and 16/06/13 to find relevant papers. These were abstract reviewed independently by two reviewers with papers meeting the inclusion criteria subjected to data extraction.

**Results:**

33 publications (32 papers and one book chapter) were included which contained 32 distinct methodologies. The majority of these methodologies (n = 22) can be classed as either dialogical or consultative, and these represent two extreme ‘poles’ of methodological orientation. Consideration of these results provoked three central questions that are central to the planning of an empirical bioethics study, and revolve around *how* a normative conclusion can be justified, the *analytic process* through which that conclusion is reached, and the *kind* of conclusion that is sought.

**Conclusion:**

When considering which methodology or research methods to adopt in any particular study, researchers need to think carefully about the nature of the claims they wish to generate through their analyses, and how these claims align with the aims of the research. Whilst there are superficial similarities in the ways that identical research methods are made use of, the different meta-ethical and epistemological commitments that undergird the range of methodological approaches adopted rehearse many of the central foundational disagreements that play out within moral philosophy and bioethical analysis more broadly. There is little common ground that transcends these disagreements, and we argue that this is likely to present a challenge for the legitimacy of the bioethical enterprise. We conclude, however, that this heterogeneity ought to be welcomed, but urge those involved in the field to engage meaningfully and explicitly with questions concerning what kinds of moral claim they want to be able to make, about normative justification and the methodological process, and about the coherence of these components within their work.

## Background

This paper reports a systematic review of methodologies related to a particular kind of empirical bioethics (EB) research – specifically, methodologies that seek to use empirical data about stakeholder values, attitudes, beliefs and experiences to inform normative ethical theorising.

‘Empirical bioethics’ is a generic and broad term increasingly used to describe a particular kind of research endeavour that seeks to ask and answer questions of bioethical interest in a way that draws on the strengths of both philosophical and empirical analysis [1-4]. Its ‘rise’ can, in part, be seen as a response to the social science critique of philosophical bioethics, as described by Hedgecoe [5], which challenges what is seen as ‘traditional’ philosophical bioethics to become more contextually aware and more grounded in the realities of lived experience. In the context of this review we are, therefore, particularly interested in looking at methodologies that seek to use social scientific data (usually about stakeholder attitudes, beliefs and experiences) to inform and enhance ethical analyses of topics of bioethical interest.

Even thus specified, the term ‘empirical bioethics’ is commonly understood to refer to a wide range of varying methodologies that have different views about how best to respond to the challenge of connecting normative bioethical analysis to the realities of lived moral experience. There is considerable uncertainty about the range and substance of these methodologies, and there remains a difficulty in trying to articulate their aims and content. One standard response has been to articulate typologies that differentiate between different kinds of research endeavour, and a number of useful typologies have, to date, been put forward to describe how philosophical theory and data can be combined [6-10]. DeVries [6], for example, offers a four-part typology that distinguishes between practical research strategies that: (i) use empirical data to describe attitudes toward an issue; (ii) use empirical data to explore the likely or actual consequences of bioethical policies and decisions; (iii) use empirical data to explore the ‘implicit normativity’ in scientific/clinical practice, and (iv) use empirical data to understand the institution of bioethics.

An alternative, and quite different, typology is offered by Molewijk *et al*. [9] , who distinguish between different kinds of research strategy by reference to the locus of moral authority in order to clarify how best to arrive at a normative conclusion. Their typology differentiates between four research strategies that: (i) give complete authority to moral theory, and only use empirical data to provide evidence for premises or support factual claims; (ii) give precedence to moral theory but accommodate a one-way relationship between theory and data such that empirical research can be used to refine theory; (iii) give equal authority to both theory and data, such that both theory and interpretation of data can be adjusted in light of the other, and: (iv) remove theory altogether from ethical analysis and focus only on the particulars, which are identified through empirical research.

These typologies of general research strategies are useful because they begin to carve out the field according to what a piece of research might seek to achieve, and helpfully distinguishes between various ways of thinking about the research endeavour. What they do not do is explore the detail of, and differentiate between, different methodologies that fall within each of those strategies. A useful addition to this field would be, therefore, a systematic review of the methodological variation within these strategies. We note that systematic reviews in the field of bioethics are relatively rare, although their use is increasingly proposed [11,12]. The rationale for conducting a systematic review, as opposed to the narrative reviews that we can find in the literature to date, are, as Strech and Sofaer point out: “to reveal a greater range of…information than the informal reviews …that are usual in bioethics and philosophy, which sample literature using unsystematic, undocumented search methods to the unspecified point at which it seems to the author (often the only author) that [nothing new emerges]” [11], p122.

This paper reports a systematic review focussing specifically on methodological strategies that seek to draw normative conclusions through the integration of social scientific empirical data collection/analysis and normative/ethical theorising. This might best be thought of as falling within Molewijk *et al's*. [9] category of both *Critical Applied Ethics* or *Integrated Ethics*, and which we describe simply as being ‘integrative’. This integrative approach seems to offer the best chance, at least in theory, of genuinely accessing the strengths of both the empirical and the philosophical contributions, and through our engagement with the field to date we felt that it is the approach that has received the most methodological attention, is the most heterogeneous, and is most in need of systematic review. In addition, the report from the first workshop of the UK based Interdisciplinary and Empirical Ethics Network (IEEN) [13], concluded that there are important questions to ask about, *inter alia*:

1. What kinds of normative conclusions empirical bioethics ought to be aiming for, suggesting that “[t]he debate over whether their research seeks objective and universalisable answers, or more subjective and particularist answers, is perhaps best worked out locally given the particular aims and agendas defined in each research project”. [13] p160.
2. The way that empirical bioethics seeks to provide justification for its conclusions, stating that “The fundamental question concerning justificatory authority - how we can articulate why and how our conclusions can be considered better, or worse, than anyone else’s – remains unresolved”. [13] p160.

Our focus on this kind of integrated empirical bioethics is, ultimately, premised on our personal interest in pursuing and developing methodologies for bioethics research that take seriously the empirical and the contextual, but which are also concerned with producing normative conclusions and justification for those conclusions. That is not to say this is the only kind of legitimate bioethics activity – only that it is the activity we are primarily concerned with and interested in. It is also a research strategy of central importance to the field of bioethics that has been well-recognised by other scholars as being in need of further development, reflection and scrutiny [14-16]. Other reviews of different kinds of empirical bioethics activity would undoubtedly be a welcome addition to the literature and give rise to different theoretical and methodological implications.

With this in mind, this review aimed to:

1. Identify the number and range of published ‘integrative’ empirical bioethics methodologies available.
2. Explore differences and similarities between those published methodologies, with a view to identifying and discussing key questions about aims, analytic process and justification that differentiate between different kinds of methodologies.

## Methods

The initial search for this review was conducted between February and March 2012. The review was conducted in accordance with the Cochrane guidelines [17]^a^. Three databases (Google Scholar, Web of Science and MedLine) were searched, up until 16/03/2012. These three databases were chosen because we found that, between them, they indexed all the kinds of journals in which our target papers were likely to be published. Given the time and resource restrictions on the project^b^, we made a conscious decision to use databases that would index likely journals; i.e. those that publish papers around the nexus of medicine, healthcare, ethics, philosophy and research methodologies.

Broad search terms were used to allow for the wide variation in terms we knew to be present in the literature, with a separate set of terms to ensure that only papers including a methodology were returned. The most significant difficulty we had lay in the fact that there is no agreed terminology used to label this kind of research endeavour, and so we aimed to develop a very wide and inclusive search strategy. Numerous scoping searches were performed using Google Scholar to select terms that were able to identify relevant papers without turning up thousands of papers that were irrelevant. The initial search terms, developed after scoping searches, were:

- (“Empirical Bioethics” OR “Evidence Based Bioethics” OR “Interdisciplinary Bioethics” OR “Empirical Ethics”)
- AND
- (Methodology* OR Method* OR Process*)

This produced approximately 1240 search results, of which 37.5% of the first 150 passed title screening.

When applied to MedLine, however, this search produced only 19 results of which 18 passed title screening. This indicated that our search terms were sensitive to keywords used on Google Scholar but not MedLine. The search was therefore extended to include additional and, arguably, less technical terms, that seemed likely to capture papers that talked about relevant methodologies without naming them. These additions increased the sensitivity of the search in Medline, and had no significant effect in Google Scholar. The final set of search terms used were:

- (“Empirical Bioethics” OR “Evidence based bioethics” OR “Interdisciplinary bioethics” OR “Empirical Ethics” OR “Social sciences perspectives on bioethics” OR “Empirical Research in Bioethics” OR “Empirical-Ethical Research” OR “”Ethics-Related Empirical Research”)
- AND
- (Methodology* OR Method* OR Process*)

Truncations were used to cover the second phase of the search in order to include all forms of the words. Both ‘methodology’ and ‘method’ were used, to allow for the fact that some authors may refer to the overriding process as a ‘method’.

No limits were placed on the publication date, meaning that any relevant papers, regardless of publication date, would be found. A small secondary search was also performed using existing bibliographies of EB literature ^c^ and by searching the references of included papers.

Search results were title screened by RD for relevance, followed by abstract review against the inclusion criteria (see List of Criteria for inclusion into review). Multiple papers by the same author could be included, as could publications not exclusively aimed at *bio*ethics if they met all other criteria^d^. Abstract review was performed independently by two reviewers (RD and JI), who rated the papers as either ‘accept’, ‘reject’, or ‘unclear’. Both reviewers met to compare their independent reviews. All abstracts for which there were concordant ‘agree’ reviews were accepted for data extraction. All papers for which there was a concordant ‘reject’ review were eliminated. In the case of disagreement, or when either reviewer was unsure, the entire paper was screened followed by the same review process. Included papers were subjected to data extraction using a pre-formed data-extraction sheet, which recorded details on aims, methods and epistemological/philosophical assumptions (see List of Headings from Data Extraction sheet).

### List of criteria for inclusion into review

- Paper or book chapter
- Written in English
- Discusses concept of ‘EB’, even if the phrase itself isn’t used
- Details a methodology
- Aims to draw normative conclusions (not just description, sociological analyses or gathering data to support factual premises in argument)
- Does not have to specifically deal with *bio*ethics if the methodology is transferrable

### List of headings from data extraction sheet

- Title of paper
- Author/s
- Name of methodology
- Stepwise procedure given/Overarching process described (tick boxes)
- Linear/Cyclical/Iterative/Other or none (tick boxes)
- Aim of the research/conclusion
- Details of data collection method
- Data analysis method given (if not provided why not/what is said about data analysis)
- Details of method for developing normative outcomes
- Philosophical commitments stated/implied
- Social scientific commitments stated/implied
- Theories in opposition and why: Author stated/reviewer
- Strengths and weaknesses- Philosophical: Author stated/Reviewer identified
- Strengths and weaknesses- Practical: Author stated/Reviewer identified
- Other notes

The extracted data were then used to categorise the methodologies, with the aim of identifying similarities and differences in the processes used and grouping them according to those similarities.. These process categories were then scrutinised to see if further sub-categories were warranted.

In May 2013 the search was repeated to update the results with a view to publication. The same search terms, databases, screening methods and inclusion criteria were used as in the initial search. This search was limited to show only results published since 2012 to minimise the number of duplications from the original search. Again, a secondary search of included papers’ references was undertaken, as well as any recent additions to EB reading list and bibliographies. Methods of review and data extraction were performed as described above.

### Interpretation and analysis

Data extraction allowed the key methodological characteristics of each paper to be identified and summarised, which helped us begin the interpretive process of categorisation. The papers were then initially categorised and sub-categorised on the basis of the methodological process they use. On occasion, multiple papers, often by the same authors, discussed the same processes and these were combined to remove duplication. One paper described three different methodologies. This resulted in the identification of 32 distinct methodologies, spread across 33 different papers.

The categorisation of papers was driven by our a *priori* focus on methodology; and our analysis was directed towards looking for methodological similarity and dissimilarity and, at least initially, informed by the workshop report on the IEEN (13), which called for more information on justification, process and aims. Our data extraction sheet mirrored differences that we had observed in the literature - for example, was the research process cyclical, linear or iterative? Whilst this helped us to structure our initial thinking, those three categories were not ultimately useful, as they did not fully capture what we felt were the key aspects of the methodologies that were included.

The categories and subcategories we ended up using were generated through a process of reflection and induction. After data extraction was complete, RD, JI and MD met to discuss how to categorise the 32 methodologies, and agreed on an initial two-way categorisation that distinguished between ‘dialogical’ and ‘consultative’ approaches, based on a fundamental difference. These two categories arose naturally from the data, as the methodologies tended to either generate normative conclusions with participants through a dialogue, or generate normative conclusions after consulting with participants in some way. We then attempted to place each methodology into one of those categories, and then considered whether those methodologies that did not clearly fit into either category provided evidence that the two categories were wrong, or that these categories needed to be extended. On closer examination, a few methodologies seemed to encompass elements of both categories, and some were clearly neither dialogical nor consultative but had nothing sufficiently in common to generate a new meaningful category. A similar reflective and inductive process was then used to examine methodologies within each category, leading to the consultative category being split into more specific groupings that shared a common and distinctive methodological characteristics.

## Results

**Figure 1:**
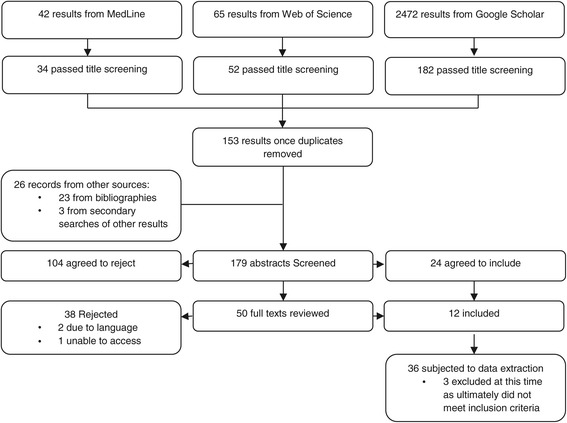
**PRISMA diagram combining both search periods.**

A total of 36 publications were initially selected for data extraction. Three were then excluded for not meeting the inclusion criteria, having been included on abstract alone, leaving 33 publications included (see Table 1). The PRISMA diagram in Figure 1 depicts the search and screening process over the two searches conducted.

**Table 1:** **Table showing included publications**, **with brief summary of methodology described**

**ID**	**Publication reference**	**Methodology**	**Summary of process described**
1	de Wachter M. **Interdisciplinary bioethics**: **But where do we start**? **A reflection on epoche as method**. *Journal of medicine and philosophy* 1982, 7(3): 275–288.	Interdisciplinary Epoche	Approach to interdisciplinary working in bioethics, not specific EB methodology
2	Hoffmaster B. **Can ethnography save the life of medical ethics**? *Social science & medicine* 1992, 35(12):1421–1431.	Ethnography	Data (ethnography) driven, non-specific integration and generation of normative conclusions
3	Ten Have H, Lelie, A. **Medical ethics research between theory and practice**. *Theoretical Medicine and Bioethics* 1998, 19(3):263–276.	Normative Ethnography	Ethnography based consultative approach, giving equal weight to data and ethical theory
4	Richardson J. **Empirical Ethics**, **Or**, **The Poverty of Ethical Analyses in Economics and the Unwarranted Disregard of Evidence in Ethics**. Working paper 120. *Centre for Health Program Evaluation*, *Monash University* 2001.	Iterative Population-based Ethics (name assigned by reviewer)	Consultative approach that tests and refines ethical principles in light of population views
5	Battin M. **Empirical research in bioethics**: **the method of**" **oppositional collaboration**. *Notizie di Politeia* 2001, 18(67): 15–19.	Oppositional Collaboration	Methodology for how researchers should approach EB as opposed to describing a research methodology
6	Martin D, Singer P. **A strategy to improve priority setting in health care institutions**. *Health Care Analysis* 2003, 11(1):59–68.	Describe-evaluate-improve (name assigned by reviewer)	Consultative approach that compares how a practice *is* with how it *ought* to be. No information given as to how it worked out what the practice ought to be
7	Borry P, Schotsmans P, Dierickx K. **What is the role of empirical research in bioethical reflection and decision**-**making**? **An ethical analysis**. *Medicine*, *Health Care and Philosophy* 2004, 7(1):41–53.	Step-wise Empirical Contributions	Proposal of how empirical data can be inputted at each stage of a typical ethical decision making process, rather than a presenting a methodology. Data is always subservient to theory
8	Molewijk B.,Stiggelbout A, Otten W, Dupois H, Kievit J. **Empirical data and moral theory. A plea for integrated empirical ethics**. *Medicine*, *Health Care and Philosophy* 2004, 7:55-69	Integrated Empirical Ethics	Broadly consultative approach which aims to achieve strong interdisciplinary cooperation and the effective dissolution of the fact/value distinction. Suggests that either reflective equilibrium or pragmatic hermeneutics might be able to achieve this.
9	Reiter-Theil S. **Does empirical research make bioethics more relevant**? “**The embedded researcher**” **as a methodological approach**. *Medicine*, *Health Care and Philosophy* 2004, 7(1):17–29.	The Embedded Researcher	Consultative approach where Information gathered by a researcher ‘embedded’ in a situation is used to inform the ethical decision from the ‘inside out’ although how exactly this is done is unclear
10	Arnason V. **Sensible discussion in bioethics**: **Reflections on interdisciplinary research**. *Cambridge Quarterly of Healthcare Ethics* 2005, 14(3):322–328.	Complementarity Thesis	Data led consultative approach which tests whether stakeholder views stand up to reason. It does not describe how normative conclusions are generated from this approach
11	Ebbesen M, Pedersen B. **Using empirical research to formulate normative ethical principles in biomedicine**. *Medicine*, *Health Care and Philosophy* 2007, 10(1):33–48.	Phenomenological Hermeneutics and Wide Reflective Equilibrium	Uses phenomenological hermeneutics to gather and interpret data (using a partly dialogical and partly consultative approach) and wide reflective equilibrium to conduct the analysis and to generate normative conclusions
12	Haimes E, Williams R. **Sociology**, **ethics and the priority of the particular**: **learning from a case study of genetic deliberations**. *The British Journal of Sociology* 2007, 58(3):457–476.	Sociology-led Phronesis (name assigned by reviewer)	Data led consultative approach where moral theory is only used to “find purchase” on the data. Unclear how normative conclusions are drawn, although it draws heavily on *phronesis*.
13	Draper H, Ives J. **An empirical approach to bioethics**: **social science** '**of**', '**for**' **and** '**in**' **bioethics research**. *Cognitie*, *Creier*, *Comportament*. 2007, 11(2):319–330.	Encounters with experience	Consultative approach which utilises reflective equilibrium to integrate empirical data and ethical theory. Early and less detailed exposition of ideas presented in a later paper (17)
14	Widdershoven G, van der Scheer L. **Theory and methodology of empirical ethics**: **a pragmatic hermeneutic perspective**. In *Empirical ethics in psychiatry*. Edited by Widdershoven G, McMillan J, Hope T, Van Der Scheer L. Gosport: Oxford University Press; 2008:23–35.	Pragmatic Hermeneutics	Process involving formation of dialogue between stakeholders and an external analysis followed by the generation hypotheses for policy, which are then put back into the dialogue and refined
15	Doorn N. **Applying Rawlsian approaches to resolve ethical issues**: **inventory and setting of a research agenda**. *Journal of business ethics* 2009, 91(1):127–143.	Wide Reflective Equilibrium and Overlapping Consensus	Method for integrating data and theory. Limited detail about how the data is gathered and analysed
16	Nikku N, Eriksson B. **Microethics in action**. *Bioethics 2006*, 2(4):169–179.	Microethics	Investigation of ‘everyday’ ethical problems. Unclear as to how this empirical data should be integrated with theory
17	Ives J. Draper H. **Appropriate methodologies for empirical bioethics**: **it**'**s all relative**. *Bioethics* 2009, 23(4):249–258.	Reflective Equilibrium based on ‘Encounters with experience’	Version of the reflective equilibrium approach which gathers data by sending the researcher into the field, and seeks a balance in which data is refined by theory and theory is refined by data.
18	Leget C, Borry P, De Vries R. ‘**Nobody Tosses a Dwarf**! ’**The Relation between the Empirical and the Normative Reexamined**. *Bioethics* 2009, 23(4):226–235.	Critical Applied Ethics	Consultative process that uses empirical data throughout to constantly reassess and refine normative outcomes (different exposition of 25)
19	Parker M. **Two concepts of empirical ethics**. *Bioethics* 2009, 23(4):202–213.	Teleological Expressivism	Consultative process which gives a prominent role to the public legitimisation of proposed policy. Limited detail about the individual steps that comprise this process.
20	Kim S, Wall I, Stanczyk A, De Vries R. **Assessing the public**'**s views in research ethics controversies**: **deliberative democracy and bioethics as natural allies**. *Journal of Empirical Research on Human Research Ethics* 2009, 4(4):3–16.	Deliberative Democracy	A dialogical approach which differs from others as it uses *lay* accounts and opinions rather than specific stakeholders. The idea of this is to avoid decisions being influenced by those with a vested interest. Limited detail given on *how* normative conclusions are drawn in this process, but explicit about the legitimacy of conclusions being derived from democratic ideals.
21	Schleidgen S, Jungert M, Bauer R. **Mission**: **Impossible**? **On empirical**-**normative collaboration in ethical reasoning**. *Ethical theory and moral practice* 2010, 13(1): 59–71.	Distinct Methodological Collaboration	A proposal that ethicists and social scientists should work together by following the traditions of their own disciplines and putting their findings together. Limited detail about the process of actually conducting the research.
22	van Delden J, van Thiel G. **Reflective equilibrium as a normative**-**empirical model in bioethics**. In *Reflective equilibrium*: Essays in honour of Robert Heeger, Edited by ven der Burg W. van Willigenburg T. Dordrecht; Springer: 1998, 251–259.	Normative Empirical Reflective Equilibrium	A version of reflective equilibrium which uses the moral intuitions of stakeholders for its empirical data
23	Widdershoven G, Abma T, Molewijk B. **Empirical ethics as dialogical practice**. *Bioethics 2009*, 23(4):236–248.	Response Evaluation Hermeneutics	Dialogical process that begins by giving a voice to the least heard group of stakeholders. The aim is to reach a ‘mutual understanding’, from which normative conclusions follow.
24	Abma T, Baur V, Molewijk B, Widdershoven G. **Inter**‐**Ethics**: **Towards an Interactive and Interdependent Bioethics**. *Bioethics* 2010, 24(5): 242–255.	Inter-ethics	Dialogical process for decision making in concrete situations. The ethicist acts as facilitator and may draw on ethical theory to enrich the dialogue.
25	Leget C, Borry P. **Empirical Ethics**: **The Case of Dignity in End**-**of**-**Life Decisions**. *Ethical Perspectives* 2010, 17(2): 231–250.	Critical Applied Ethics	Consultative process that uses empirical data throughout to constantly reassess and refine normative outcomes (different exposition of 18)
26	Frith L. **Symbiotic empirical ethics**: **a practical methodology**. *Bioethics 2012*, 26(4):198–206.	Symbiotic Empirical Ethics	Five step consultative approach aiming to refine and develop ethical theory, based on a naturalistic ethics that sees practice and theory as symbiotically related and mutually informing.
27	De Vries M, van Leeuwen E. R**eflective equilibrium and empirical data**: **third person moral experiences in empirical medical ethics**. *Bioethic*s 2010, 24(4):490–498	Reflective Equilibrium: Network Model with third person moral experiences	Variation of wide reflective equilibrium designed to help solve ethical problems in concrete situations. Third person experiences are inputted into the equilibrium with relevant moral theory
28	Hunt M, Carnevale F. **Moral experience**: **a framework for bioethics research**. *Journal of medical ethics* 2011, 37(11):658–662.	Moral experience hermeneutics (name assigned by reviewer)	Methodology designed to understand the moral practice of a population and therefore does not fully explore how to generate normative conclusions.
29	Landeweer E, Tineke A, Widdershoven G. **Moral margins concerning the use of coercion in psychiatry**. *Nursing ethics* 2011, 18(3):304–316.	Dialogical hermeneutics for enhanced stakeholder understanding/Inter-ethics	Dialogical approach that is very similar to *inter*-*ethics* (24)
30	Schicktanz S, Schweda M, Wynne, B. **The ethics of** ‘**public understanding of ethics**’—**why and how bioethics expertise should include public and patients**’ **voices**. *Medicine*, *Health Care and Philosophy* 2012,15(2):129–139.	Ethics of public understanding	Proposed tool for making decisions that are congruent with the views of those affected. Uses a consultative approach to gather data but is unclear about how this is analysed and used to make decisions
31	Dunn M, Sheehan M, Parker M, Hope T. **Toward methodological innovation in empirical ethics research**. *Cambridge Quarterly of Healthcare Ethics* 2012, 21(4):466–480.	Grounded moral analysis	A constructivist account that aims for full integration of empirical analysis and normative analysis to develop normative claims that are justified and that have real-world purchase. Methodology centres around an iterative process of empirical research and theory being used to influence and change each other until a normative outcome is shaped by participants
		Moral conversation	A dialogical approach coming from the same theoretical standpoint as above. Intended to bring about focussed engagement and reflection within practice by forming a dialogue between stakeholders
		Moral participation	Again, from the same theoretical standpoint as above. The researcher actively experiences a situation and then undergoes a process of critical reflection which must stand up to ethical reasoning
32	Rehmann-Sutter C, Porz R, Leach Scully J. **How to Relate the Empirical to the Normative**. *Cambridge Quarterly of Healthcare Ethics* 2012, 21(4):436–447.	Phenomenological Hermeneutics	The researcher produces ethical arguments which, on phenomenological foundations, the data provides the conditions to evaluate. Involves hermeneutic ‘circles’- including whether the future ‘reader’ of any conclusion made will consider their findings to have any normative authority
33	Ives J. **A method of reflexive balancing in a pragmatic**, **interdisciplinary and reflexive bioethics**. *Bioethics* 2014. 28(6):302–312.	Reflexive Balancing	Although utilising the concept of coherence this methodology is distinct from reflective equilibrium in that it gives initial weighting to certain ‘boundary’ principles with which coherence is sought but which must then justify their own inclusion. This is likened to a ‘null hypothesis’ which must be proven or disproven. An explicitly pragmatic process, relying on a naturalistic ethics that focuses on achieving a defensible compromise in genuinely dilemmatic situations.

**Figure 2:**
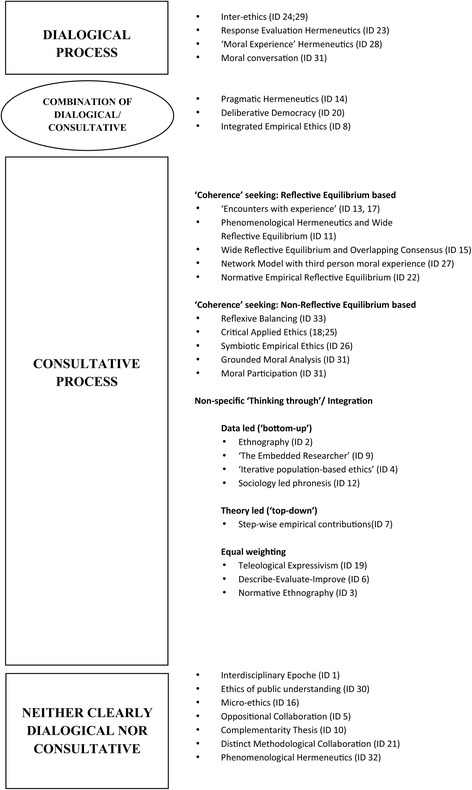
**Categories illustrating methodological processes.**

Figure 2 (below) illustrates the way the included methodologies were categorised. The majority of the methodologies outlined in these publications (n = 22) can be classed as either dialogical or consultative, and these represent two extreme ‘poles’ of methodological orientation. Three methodologies appear to use a combination, or could use either. On one pole we find Dialogical approaches, which are based around the formation of a dialogue between stakeholders and the attempt to reach a shared understanding, in which the analysis, and reaching of a conclusion, is undertaken by the researcher and participants together. These approaches generally aim to find a resolution to a discrete problem. On the other pole we find Consultative approaches, which tend to utilise an external ‘thinker’ who gathers data and analyses it independently of the data collection process, and then develops normative conclusions. Essentially, this approach ‘consults’ with participants to obtain their views and experiences, but participants do not take part in the process of forming a normative conclusion. In consultative approaches the data can be gathered in any number of ways and may come in many different forms, and the aims of consultative approaches, vary; ranging from theory development to the generation of concrete answers to discrete problems.

The consultative group was highly varied and could be further sub-categorised according to the process used by the ‘thinker’ (or the researcher). Within the consultative group, five methodologies propose a form of Wide Reflective Equilibrium (RE), and five describe non-RE coherence based methodologies. Eight methodologies were less clear about the consultative process (or method) of drawing normative conclusions - describing a non-specific ‘thinking- through’ (our term). Within this latter group there is considerable variation in where the locus of authority is placed. Four give data priority over theory, one gives theory priority over data, and three give equal weight to both.

Finally, seven methodologies are neither clearly dialogical nor consultative, and three methodologies (pragmatic hermeneutics, deliberative democracy and integrated empirical ethics) may comprise elements of both.

## Discussion

The two poles of methodological orientation that we have used to categorise the majority of methodologies are useful in the sense that they provide a very general way of thinking about how a normative conclusion can be reached. In Dialogical approaches, the ethical analysis and reaching of a normative conclusion is part of the research encounter itself. Reaching consensus, in one form or another, plays a significant role here, with moral authority generated, for example, through a hermeneutic ‘fusing of horizons’ [18], or democratic mandate [19]. By contrast, consultative approaches involve explicit engagement with stakeholder views, but with the ethical analysis being undertaken post-engagement, by the researcher or the research team. In these latter accounts, stakeholders feed into the ethical analysis, but are not involved in it directly. Moral authority, in these approaches, is more likely to come from theoretical soundness.

Distinguishing between these two poles of methodological orientation fails, however, to accommodate the significant differences within the orientations themselves. The consultative orientation, in particular, includes a wide range of divergent methods which assume different epistemological and meta-ethical commitments. What this variation illustrates, quite strikingly, is not only the wide variation in methods, but also the attendant variation in the aims and epistemologies associated with these methods. Different methodologies produce different kinds of knowledge, such that the epistemological status of the conclusions arising out of, for example a project using ‘inter-ethics’ will be quite different from a project using ‘deliberative democracy’, ‘symbiotic bioethics’ or a ‘network model with third person moral experience’. Consequently, how normative conclusions are justified – the source of moral authority - will be different in each, which will affect the grounds upon which any conclusions can be accepted.

Considering the results of the systematic review, we feel that there are three further questions that are immediately provoked by the data presented above and the broad differences we have observed across the methodologies identified. These questions revolve around *how* a normative conclusion can be justified, the *analytic process* through which that conclusion is reached, and the *kind* of conclusions that are sought. In the remainder of this paper we outline those questions and discuss, briefly, their relevance and importance.

### *How* a normative conclusion can be justified: can moral justification be found through consensus or coherence?

This question mirrors a general philosophical debate around what justifies claims to moral knowledge, or to knowledge in general. In-depth engagement with this question may require immersion in philosophical and sociological epistemology, philosophy of science and theories of truth, which we do not have space for in this paper. The main point we are trying to make, here, is that there is a long tradition of philosophical thought that deals with these questions of justification, knowledge and truth, and a commitment to any specific empirical bioethics methodology is likely to involve aligning oneself with a particular epistemology about how a claim to moral knowledge can be justified.

A research method that appeals to consensus to justify a normative conclusion finds moral authority in agreement of some kind. Some ‘dialogical’ methods rely on hermeneutic philosophy to explain how this agreement works, appealing to a ’fusion of horizons’ premised on achieving a shared understanding and interpretation of the world. These methods rely, broadly speaking, on accepting the view that a process of dialogue can lead to people understanding the world in the same way, which leads to agreement on the correct solution. Many dialogical methods are based on facilitating that dialogue e.g. [18], and that facilitation comprises the empirical and ethical research project. The normative conclusion generated through that dialogical process becomes the findings of the research. Alternatively, some approaches seek a different kind of consensus: one based on a political philosophical claim about democratic authority rather than a meta-ethical claim about shared interpretation and moral knowledge [19]. Agreement, here, is not the basis of justification but, rather, justification flows from the legitimacy of the democratic processes invoked to draw normative conclusions.

Conversely, a research method that appeals to coherence finds moral authority, broadly speaking, in rationality and consistency. The difficult question that needs an answer is what ‘coherence’ means, what it looks like, and what has to ‘cohere’ with what. One option is coherence with moral theory, and a conclusion is coherent if it fits, logically, with a particular theoretical viewpoint. This is one traditional (analytic) philosophical approach, which an integrative empirical bioethics would tend to reject due to the lack of attention it accords to the contingent features of ‘real-world’ settings. An alternative option is that coherence may be found between data and theory (as in narrow reflective equilibrium) or between a number of relevant considerations (as in wide reflective equilibrium). A research method that draws on coherence will tend to involve some process of balancing, where a coherent position is found between the various relevant considerations.

Many of the consultative approaches identified are based on coherence, the majority of which explicitly refer to wide reflective equilibrium, and seek to find a balance (an ‘equilibrium’) between, for example: background theories, moral principles, morally relevant facts, moral experiences of others, considered moral judgements of the ‘thinker’ e.g. [20]. This method gives all the considerations listed equal weighting, with none having greater epistemic authority than any other. An alternative methodology that draws on coherence, reflexive balancing [1], rejects the notion that all considerations have equal epistemic status and uses empirical data to posit ‘quasi-foundational moral principles’ around which coherence is built; where coherence is sought between mutually supporting principles and claims. In yet another contrasting position, the three methodological approaches put forward by Dunn et al. [4] interpret coherence with reference to argumentative standards in ‘real-world’ practical reasoning, rather than in terms of any alignment between moral theory and empirical facts. Here, the purpose of the research is to develop normative claims in which there is coherence between universal standards of justification for convincing arguments of all kinds, and relevant contextual considerations that enable agents to be convinced to act in line with the requirements of these arguments.

### The *analytic process* through which that conclusion is reached: should we prioritise the thinker, the theory or the stakeholders?

One of the most fundamental questions that must be considered is who, or what, should be doing the substantive analytic work in empirical bioethics research. Broadly speaking, in the methods and methodologies identified above we find three possible answers to this question.

First, a methodology where the thinker does the work is reliant upon a single central person (or sometimes central team). This would usually be the researcher, or the research team, who takes the analytic burden and whose judgement is central to the analytic process. Any normative conclusions belong to that ‘thinker’ (or group of thinkers), and a different, but similarly robust and coherent, thinker (or thinkers) could reach a different conclusion, and this may impact on how the conclusions are presented, requiring, perhaps, a certain amount of reflexivity see [21]. In our sample of included methodologies those that prioritise the thinker tend to be consultative, and these include all the methodologies that draw on reflective equilibrium.

Second, a methodology that prioritises the theory still requires a person to conduct the analysis, but the focus is on the logical and consistent development and application of theory, such that the theory dominates the normative analysis. This kind of method wants to generate conclusions that, once the theory is agreed upon, would be binding for all rational agents (or, at least, all rational agents who subscribe to that theory, thus expressed). In doing this, the ‘correct’ theory is identified in advance, and one’s normative conclusions would be the result of how the theory is applied, given the contingencies of the empirical data that is taken into account. It is noteworthy that some methodologies, for example Frith’s ‘symbiotic bioethics’, attempt to use data to help refine theory, and then apply that more contextualised theory to the problem in hand (rather than deciding on the theory in advance of the research process). Other methodologies, such as Borry *et al*’*s* ‘step wise empirical contributions’ [22] aim to input empirical data into various stages of the ethical decision making process, but use the data to support argument defined by theory that is not subject to revision after considering the data.

Third, a methodology that prioritises the stakeholders does away with the central role of the thinker or theory. Instead, the analysis is the product of a group process that connects a broad range of voices to link relevant practical experiences to ethical considerations in a range of differently structured facilitative processes (e.g. discussion groups, interviews). In doing this, the role of the researcher is less central, and becomes more one of facilitation and process than substantive analytic contribution – such as in the dialogical methods identified in our review. The researcher does not generate or own the normative conclusions, but rather discovers and communicates the conclusions reached through the research process (whether that is stakeholder dialogue, democratic consensus, or any other kind of process).

### The *kind* of conclusion that is sought: should we aim for particularity or generalisability?

The extent to which we might want our conclusions to be particular to a specific time and context, or more universally applicable, will have a significant bearing on the method we choose. Methodologies that prioritise abstract theory may have a better claim to drawing universalisable conclusions than methods that tie an analysis to a specific dataset or a specific group of people. Methodologies that prioritise the particular [23], for example, or which seek a dialogical consensus between a specific group of people, may have difficulty drawing conclusions that go beyond that particular problem or context. One way of understanding such approaches may be to frame them as action research, with the specific aim of evaluating and changing a discrete area of practice rather than making general claims about ethics. Other methods, for example [2], explicitly aim to use empirical studies to inform and develop generalisable theory, which can then be applied in other contexts.

If we wish to conduct research that is focussed on a very specific problem, and are not concerned with making generalised moral claims, we need a method that will accommodate this narrow focus. Similarly, if we wish to be able to make universal moral claims, we need a method that either accommodates large scale generalisible empirical research, or that has a process for moving from the particular to the generalisible by way of theory generation, and which has an attendant meta-ethics that we can defend (or at least explain). This question, and the one above, tracks very closely debates in moral philosophy over particularism vs. universalism, and the debate around moral relativism. These debates are concerned respectively with whether or not one can make moral claims that apply universally, and whether or not a moral judgement can only be correct relative to the context about which it is made or the person who made it. Clearly, however one answers the two methodological questions above, one is making a claim about these debates, and making a statement about which meta–ethical view is correct.

### Limitations

Before drawing our final conclusions, we need to reflect on the limitations of this review. First, while our search strategy was robust, it is likely that some relevant papers have been missed because our search terms were not as refined and focused as they might have been. The range of different terminology used in this field is significant, and our choice of search terms was based on our existing familiarity with the literature. It is possible that there are papers in existence that are highly relevant that nonetheless use terminology that we are not familiar with. Should those papers exist, our search terms are unlikely to have captured them.

Second, we have been unable to include, or search for, non-English papers, meaning that our review is limited to English language publications. We are aware that there are groups and individuals working on empirical bioethics methods across Europe and beyond, and we have only been able to capture their English language papers. Many people working on this topic may not have published in English at all, and it is a limitation that this work could not be included.

Third, and finally, our analysis and subsequent classification is based on our own (systematic) interpretation of the field, and cannot be considered to be the last word. We feel that our categorisation of the papers identified in the review offers a useful way to look at the strategies, methods and methodologies in empirical bioethics, and through that we have highlighted some specific questions that we feel are important to consider when choosing a methodology. However, the questions we have interrogated are by no means exhaustive or defining of the field.

## Conclusion

It is important to recognise that the questions we have asked above, about consensus/coherence, particularity/universalizability, or who/what takes analytic priority, are all interconnected. They concern central questions of normative justification that no empirical ethicist can afford to ignore, and have significant implications for the design of empirical ethics studies. If one is going to design an empirical ethics study correctly, methodological decisions must be responsive to the different theoretical positions that underpin alternative approaches to empirical ethics research. When considering which methodology or research methods to adopt in any particular study, researchers need to think carefully about the nature of the claims they wish to generate through their analyses, and how these claims align with the aims of the research. In disseminating these claims, moreover, researchers must also be able to articulate and defend one or more of these positions in order to convince their audiences about the veracity and scope of the normative conclusions they draw.

The pressing need to engage with these kinds of question is highlighted by increasing calls for more public engagement in policy relevant ethical deliberation. A good example of this is the recent Nuffield Council on Bioethics working party on emerging biotechnologies, which calls for a ‘public discourse ethics’. This report [24], in chapter 4, discusses the virtues and benefits of engaging the public in these policy debates, such as candour, sustainability, equity, accountability (mirroring the kind of language used to talk about patient and public involvement in health and policy research more generally), but does not consider how this can be achieved, and seems to take for granted that it is (a) possible and (b) methodologically unproblematic.

The observations made in this paper highlight the fact that this kind of methodology is anything but unproblematic. Even within the relatively narrowly defined ‘integrative’ approaches to empirical bioethics that we have focused on, there is little in common between the various traditions of empirical ethical enquiry that we have identified. Whilst there are superficial similarities in the ways that identical research methods are made use of, the different meta-ethical and epistemological commitments that undergird the range of methodological approaches rehearse many of the central foundational disagreements that play out within moral philosophy and bioethical analysis more broadly. There is little common ground that transcends these disagreements, and the disparate nature of the methodological literature arguably makes it difficult to view empirical bioethics as a coherent (and ultimately legitimate) research enterprise. It has not developed as a cohesive body of work with a shared intellectual heritage and agreed standards, as one might see in an established discipline or methodological tradition. Rather, it has emerged as a body of work comprising distinct and entirely bespoke methodological responses to a ‘problem’ that is not always clearly defined, or understood in the same way.

In our view, the heterogeneity we have observed is not a problem in itself. Difference adds to the richness of the field and, certainly in its infancy, a field such as empirical bioethics will surely benefit from experimentation and variety. The methodological and epistemological differences we have seen, even within the narrow scope of ‘integrative’ approaches, are no more trenchant than the differences we might observe, for example, within the social sciences, and the differences seem to reflect legitimate theoretical disagreements over the purpose and nature of ethical enquiry.

This does, however, make it difficult, if not impossible, to critically assess a piece of empirical bioethics work with reference to anything outside of the work itself. The fact that the emerging literature tends to focus on developing and articulating methodologies that are bespoke to particular sets of questions, in a particular context, makes it difficult to look at a piece of work from an external perspective and assess its quality, because all of the justificatory work has to be done almost from scratch to validate that piece of work. The nature of this (early) work is that it is experimental, creative, and crosses disciplinary boundaries, and there is not an established canon of literature that can be referred to. A pioneer, if we might use that analogy, has to go it alone.

When we consider these characteristics alongside the requirements of funding and publication, and the (understandable) imperfections of peer review, it is not difficult to see how work in empirical bioethics could struggle to find a place. The shortcuts that can be taken when explaining and justifying work undertaken within clear disciplinary silos are not available to empirical bioethics. There is no standard approach to cite, there is no accepted methodology or set of methods to fall back on, and the process of offering justification for every methodological choice from first principles takes a lot of space, which is rarely available.

One way forward may be to attempt to find some level of consensus on what is required of an empirical bioethics methodology, and what standards we might use to assess the quality of work proposed and/or undertaken under this broad umbrella. Establishing a consensus that outlines areas of agreement may be able to provide some external and relatively concrete validation for at least some kinds of work. Such areas of agreement might include, for example: what assumptions one may legitimately make, and whether the theoretical assumptions behind one’s approach need to be stated, explained or fully justified (from first principles).

Our concern about that kind of solution (aside from the, perhaps obvious, difficulty of reaching a consensus), is that the field may not be mature enough to put any of these questions to bed, and given that it would be damaging to the legitimacy of the empirical bioethics endeavour to try to avoid them.

For now, our view is that everyone working in this field must live with a great deal of uncertainty, and will have to work hard to explain what they are doing and why it ought to be taken seriously. If we are trying to do a new kind of ethics, using new kinds of methodologies, then we should be put under pressure to justify and articulate that new approach clearly. At this stage in the development of empirical bioethics, that means engaging explicitly and meaningfully with questions concerning the kinds of moral claim that empirical bioethicists want to be able to make, about normative justification and the methodological process, and about the coherence of these different components of their work. So long as this is done, the evident heterogeneity doesn’t matter, and should in fact be welcomed.

## Endnotes

^a^The Cochrane handbook is intended to be used for systematic reviews of interventions. In light of this not all stages were relevant.

^b^The project was carried out as an undergraduate student project, with a strict time limit and limited resources.

^c^Bibliographies were supplied by JI and MD, which were generated for teaching purposes.

^d^Background reading suggested a body of literature on methodologies within business ethics which could also be relevant to this research.

## Abbreviations

EB: Empirical bioethics
RE: Reflective equilibrium
